# On the pixel selection criterion for the calculation of the Pearson's correlation coefficient in fluorescence microscopy

**DOI:** 10.1111/jmi.13273

**Published:** 2024-02-13

**Authors:** Sergio G. Lopez, Sebastian Samwald, Sally Jones, Christine Faulkner

**Affiliations:** ^1^ Cell and Developmental Biology The John Innes Centre Norwich Research Park Norwich UK

**Keywords:** biomolecule interactions, colocalisation, fluorescence, microscopy, Pearson's correlation coefficient

## Abstract

Colocalisation microscopy analysis provides an intuitive and straightforward way of determining if two biomolecules occupy the same diffraction‐limited volume. A popular colocalisation coefficient, the Pearson's correlation coefficient (PCC), can be calculated using different pixel selection criteria: PCC_ALL_ includes all image pixels, PCC_OR_ only pixels exceeding the intensity thresholds for either one of the detection channels, and PCC_AND_ only pixels exceeding the intensity thresholds for both detection channels. Our results show that PCC_ALL_ depends on the foreground to background ratio, producing values influenced by factors unrelated to biomolecular association. PCC_AND_ focuses on areas with the highest intensities in both channels, which allows it to detect low levels of colocalisation, but makes it inappropriate for evaluating spatial cooccurrence between the signals. PCC_OR_ produces values influenced both by signal proportionality and spatial cooccurrence but can sometimes overemphasise the lack of the latter. Overall, PCC_AND_ excels at detecting low levels of colocalisation, PCC_OR_ provides a balanced quantification of signal proportionality and spatial coincidence, and PCC_ALL_ risks misinterpretation yet avoids segmentation challenges. Awareness of their distinct properties should inform their appropriate application with the aim of accurately representing the underlying biology.

## 1 INTRODUCTION

One of the most frequent questions in biology concerns the spatial relationship between biomolecules. It is often necessary to determine if two biomolecules occupy the same diffraction‐limited space within the cell, in many cases as a preliminary step to the implementation of more specific studies aimed at testing for biomolecule–biomolecule interaction, such as those relying on Förster Resonance Energy Transfer (FRET).^1^, ^2^, ^3^, ^4^ The colocalisation analysis of fluorescence microscopy images is an easy‐to‐implement methodology that provides quantitative information about the extent to which two biomolecules occupy the same space.^5^, ^6^, ^7^ Fluorescence colocalisation does not require costly specialised equipment; all that is needed for its implementation is a standard confocal microscope. This makes fluorescence colocalisation more affordable and accessible than other light microscopy techniques commonly used to study biomolecular interactions, like Fluorescence Cross‐Correlation Spectroscopy (FCCS)^8^, ^9^ and Image Cross‐Correlation Spectroscopy (ICCS).^10^, ^11^ Although the latter techniques and techniques based on FRET are arguably the most appropriate methodologies to prove that two biomolecules are indeed interacting with each other, there are other scenarios in which fluorescence colocalisation is the only feasible option. For instance, FRET can only occur if the fluorophores used to label the biomolecules are less than 10 nm apart from each other.^12^ Furthermore, if the fluorophores are large molecules like, for example, fluorescent proteins, they will be unable to sample all possible orientations during the excited lifetime of the donor fluorophore.^13^, ^14^ Under these conditions of relative immobility, the orientation of the fluorophores becomes a critical and potentially limiting factor in FRET. If the fluorophore orientations are not appropriate, FRET will not take place, regardless of the distance that separates the fluorophores.^12^ FCCS relies on point measurements and is thus unsuited to studying biomolecular interactions over large regions of the cell.^8^, ^9^ ICCS and FCCS can only be applied within a limited range of fluorophore concentrations, which may not coincide with the naturally occurring range of concentrations of the biomolecules under study. Finally, molecular crowding within the cell can cause ICCS and FCCS data to be heavily influenced by anomalous diffusion, making it difficult to extract the parameters of interest.^15^, ^16^ In contrast, fluorescence colocalisation can be applied to variegated specimens, including both live and fixed samples, in a wide range of fluorophore concentrations, and causing less photodamage to the cell than with other techniques.

Fluorescence colocalisation relies on spatial information rather than a change in molecular properties such as occurs in FRET. However, it is a diffraction‐limited methodology and cannot be used to prove that two biomolecules are interacting with each other. The reason for this is that the diffraction‐limited volume can accommodate thousands or even millions of fluorescently labelled molecules. Hence, the cooccurrence of the two fluorescent signals within such a volume cannot be said to be evidence that the molecules are located close enough to each other to be interacting. Having said that, colocalisation metrics that quantify not only the spatial coincidence between the signals but also the relationship between the signal intensities are more likely to reveal real molecular interaction. One of these metrics is the Pearson's correlation coefficient (PCC), which can adopt values that range from −1 (mutual exclusion of the biomolecules) to 1 (perfect colocalisation of the biomolecules). A PCC value close to zero indicates no relation between the two signals.^17^ Albeit green and red are the colours that have typically been used to refer to the two images involved in the calculation of PCC, when displaying the images we have chosen here to replace the red colour with magenta to aid visualisation by colour blind readers. For historical reasons and for the sake of clarity, however, we will still refer to the two images as the ‘green’ and ‘red’ images.

Despite the popularity of the PCC coefficient, there are details about its implementation that remain shrouded in ambiguity. For instance, not much attention has been paid to the criterion employed to select the pixels that will be included in the calculation. However, as will be shown later, the pixel selection criterion exerts a significant influence on the absolute magnitude of the coefficient. In addition, it impacts the dependency of this coefficient on parameters such as the degree of overlap between objects and the ratio of foreground to background pixels. To our knowledge, three different pixel selection criteria have been adopted by the software packages now in circulation, resulting in three fundamentally different Pearson's correlation coefficients, PCC_ALL_, PCC_AND_ and PCC_OR_. In the case of PCC_ALL_, all pixels in the image are included in the calculation, regardless of their signal intensity. Therefore, even background pixels have an effect on the magnitude of PCC_ALL_. On the other hand, PCC_AND_ and PCC_OR_ rely on the application of thresholds to the red and green images in order to take only foreground pixels into account. PCC_AND_ and PCC_OR_ differ on the type of foreground pixels that are considered in each case. PCC_AND_ includes in the calculation pixels that are above both the red and green thresholds, whereas PCC_OR_ includes in the calculation pixels that are either above the red or the green threshold. PCC_OR_ also includes, like PPC_AND_, pixels that are above both thresholds.

Here we present an analysis of how the pixel selection criterion impacts the magnitude and behaviour of PCC. We include illustrative examples designed to raise awareness of the limitations and advantages of each of the pixel selection criteria. We examine the influence of the foreground to background ratio and of the degree of overlap between objects and use the outputs to provide advice on which criterion to choose depending on the characteristics of the specimen, and on how to best acquire the images in order to avoid analysis pitfalls.

## 2 MATERIALS AND METHODS

### 2.1 Confocal imaging

A commercial *Convallaria majalis* sample (Leica Microsystems) stained with safranin O and fast green FCF was imaged on a Stellaris 8 FALCON upright confocal microscope (Leica Microsystems) using an HC Plan Apo 10× air objective with a numerical aperture of 0.40 (Leica Microsystems). The sample was excited with the 514 and 620 nm outputs of a pulsed SuperK Fianium FIB‐12 PP laser source (NK Photonics) working at an 80 MHz repetition rate. Hybrid X detectors (Leica Microsystems) were employed to collect the fluorescence of the sample in the 529–570 nm (safranin O) and 635–687 nm (fast green FCF) ranges. The pinhole was set to one Airy unit at 540 nm (diameter = 49.4 μm). The voxel size was 2.28 by 2.28 by 2.00 μm in *x*, *y* and *z*, respectively. The z‐stack comprised 251 z‐slices spanning a 500 μm range. The step size used for the z‐stack (2.00 μm) complied with the requirements imposed by the Nyquist–Shannon sampling theorem.^18^

### 2.2 2 Image processing and analysis

All statistical testing, image processing, and image analysis were done in Python (Anaconda software distribution, version 3.11.4., https://anaconda.com) using the Numpy,^19^ scikit‐image,^20^ Matplotlib,^21^ Seaborn^22^ and Pandas^23^ libraries. Mock images featuring two‐dimensional Gaussian functions and overlapping squares were also generated and analysed in Python using the aforementioned libraries. The Python code can be found on GitHub (https://github.com/SergioGabrielLopez/PixelCriterionColocalization).

### 2.3 Definition of the PCC coefficients

The PCC coefficients that result from the application of the PCC_ALL_, PCC_AND_, and PCC_OR_ pixel selection criteria are defined as

$$PCC\;=\;\frac{\sum \left(R_{i}-R_{aver}\right)\cdot \left(G_{i}-G_{aver}\right)}{\sqrt{\sum {\left(R_{i}-R_{aver}\right)}^{2}\cdot \sum {\left(G_{i}-G_{aver}\right)}^{2}}},$$

$$G_{aver}=\frac{\sum G_{i}}{\left|\left\{G_{i}\right\}\right|}\;\;\mathrm{and}\;\;R_{aver}=\frac{\sum R_{i}}{\left|\left\{R_{i}\right\}\right|},$$

$$PCC\;=PCC_{ALL}\;\;\mathrm{if}\;\;R_{thresh}=\;0\;\mathrm{and}\;\;G_{thresh}=\;0,$$

$$\begin{array}{ccc} PCC & = & PCC_{AND}\;\;\mathrm{if}\;R_{thresh}>0\;\mathrm{and}\;G_{thresh}\;>0\;\mathrm{and}\;R_{i}>\\ & & R_{thresh}\;\mathrm{and}G_{i}>\;G_{thresh}, \end{array}$$

$$\begin{array}{ccc} PCC & = & PCC_{OR}\;\mathrm{if}\;R_{thresh}>0\;\mathrm{and}\;G_{thresh}\;>0\;\mathrm{and}\\ & & R_{i}>\;R_{thresh}\;\mathrm{and}/\mathrm{or}\;G_{i}>\;G_{thresh}, \end{array}$$

where $G_{i}$ and $R_{i}$ are the intensities of pixel i in the green and red images, respectively, and $G_{thresh}$ and $R_{thresh}$ are the intensity thresholds applied to the green and red images.

## 3 RESULTS

**FIGURE 1:**
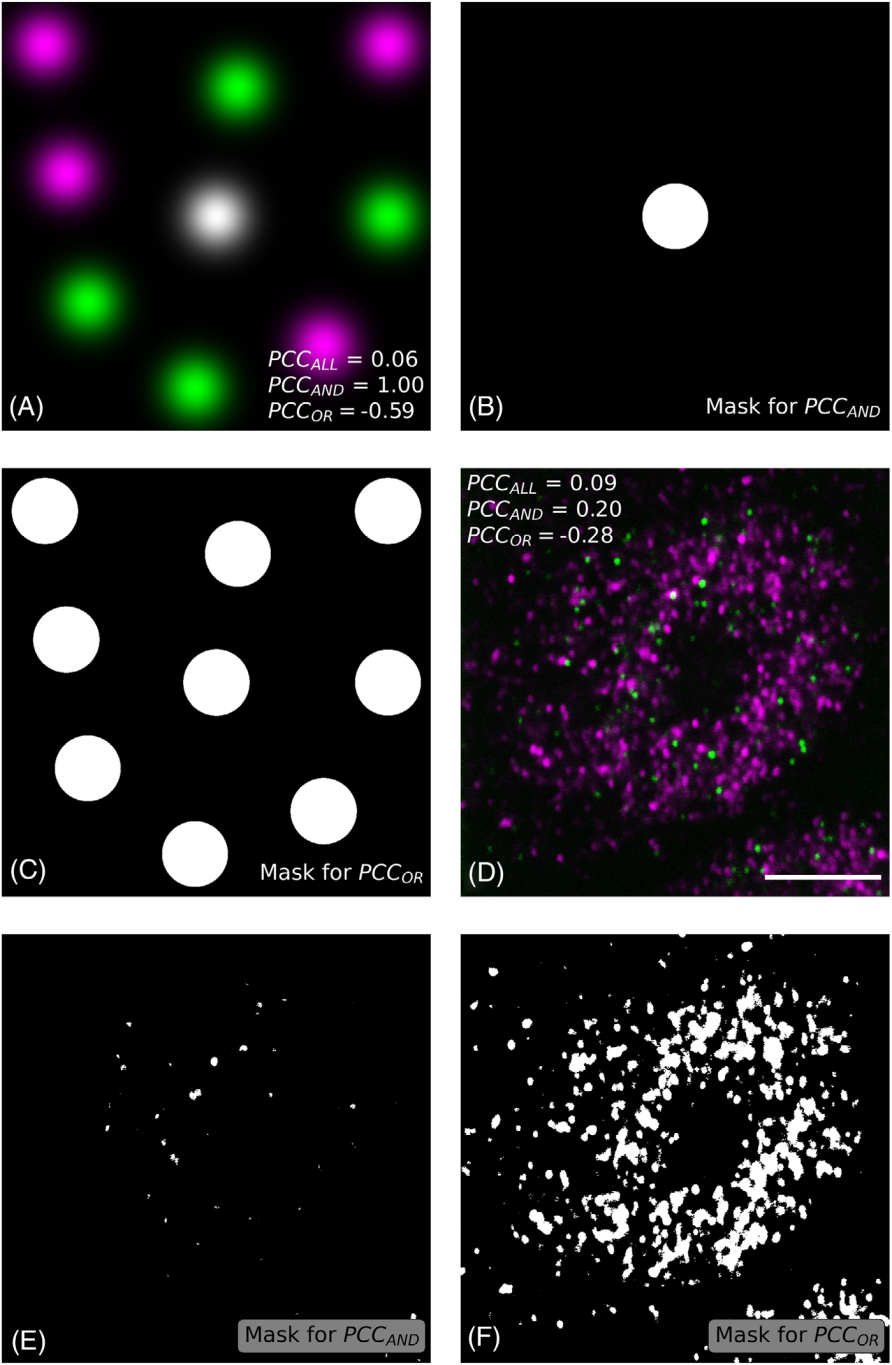
Effect of the pixel selection criterion on the magnitude of the PCC value. (A) Overlay of magenta and green images featuring two‐dimensional Gaussian functions of identical diameter and intensity on a dark 1000 × 1000 pixel backdrop. The image display is nonlinear with a Gamma factor of 2. The white colour indicates overlap between the magenta and green signals. (B), (C) The pixels in white were included in the calculation of PCC_AND_ and PCC_OR_, respectively, after application of the Otsu thresholding method to the image shown in (a). (D) Colocalisation between BRCA1 (Alexa Fluor 488, green) and Smad3 (Alexa Fluor 568, magenta) in HEK293 cells. The white areas are areas in which the green and magenta signals colocalise. This image was downloaded from the EMBL‐EBI BioImage Archive^26^ and corresponds to figure 2e (left) in reference Kawai and Amano.^25^ Bar, 5 μm. (E), (F) The pixels in white were used for the calculation of PCC_AND_ and PCC_OR_, respectively, for the image shown in (D).

Even when applied to the same image, different pixel selection criteria can result in differences in the magnitude of PCC. We produced an artificial image in which 5 objects of equal intensity and diameter were generated from two‐dimensional Gaussian functions in each collection channel, with one object from each channel placed to overlap perfectly (Figure 1A). We applied the Otsu thresholding method^24^ to select pixels based on different PCC criteria. For PCC_ALL_, every pixel in the image is selected. As is evident from Figure 1B and C, the different criteria inherent in calculations of PCC_AND_ and PCC_OR_ result in the selection of markedly dissimilar sets of pixels. In the case of PCC_AND_ (Figure 1B), only the area around the central 2D Gaussian function in which the object from both collection channels overlaps is included in the calculation, whereas in the case of PCC_OR_ (Figure 1C), areas surrounding every 2D Gaussian function are included in the calculation. The percentage of the pixels that are selected in each case is 100%, 17%, and 2% for PCC_ALL_, PCC_OR_, and PCC_AND_, respectively. The PCC_ALL_, PCC_AND_ and PCC_OR_ coefficients for the image shown in Figure 1A are 0.06, 1.00 and −0.59. This is a striking result, considering that the first value is consistent with no relation whatsoever between the two signals, the second value suggests the strongest degree of colocalisation possible, and the third value is indicative of mutual exclusion. Similar inferences can be drawn from the analysis of Figure 1D, which is a fluorescence micrograph showing BRCA1 (green, Alexa Fluor 488) and Smad3 (magenta, Alexa Fluor 568) proteins in HEK293 cells. This image was first published elsewhere^25^ and was made available via the EMBL‐EBI BioImage Archive.^26^ As with Figure 1B and C, the images shown in Figure 1E and F were both obtained using the Otsu thresholding method and any difference between them is attributable solely to the pixel selection criterion applied in each case. The percentage of pixels included in the calculation is 100%, 13% and 0.2% for PCC_ALL_, PCC_OR_ and PCC_AND_. The corresponding PCC_ALL_, PCC_AND_ and PCC_OR_ coefficients are 0.09, 0.20, and −0.28, respectively. Once again, the values differ significantly and could be considered suggestive of completely opposing scenarios. Figure S1 shows the cytofluorograms that are obtained by applying the PCC_ALL_ (Figure S1A), PCC_OR_ (Figure S1B) and PCC_AND_ (Figure S1C) pixel selection criteria to the image shown in Figure 1D. The slopes of the regression lines displayed in Figure S1A–C facilitate the understanding of why PCC can sometimes take a positive or negative value.

**FIGURE 2:**
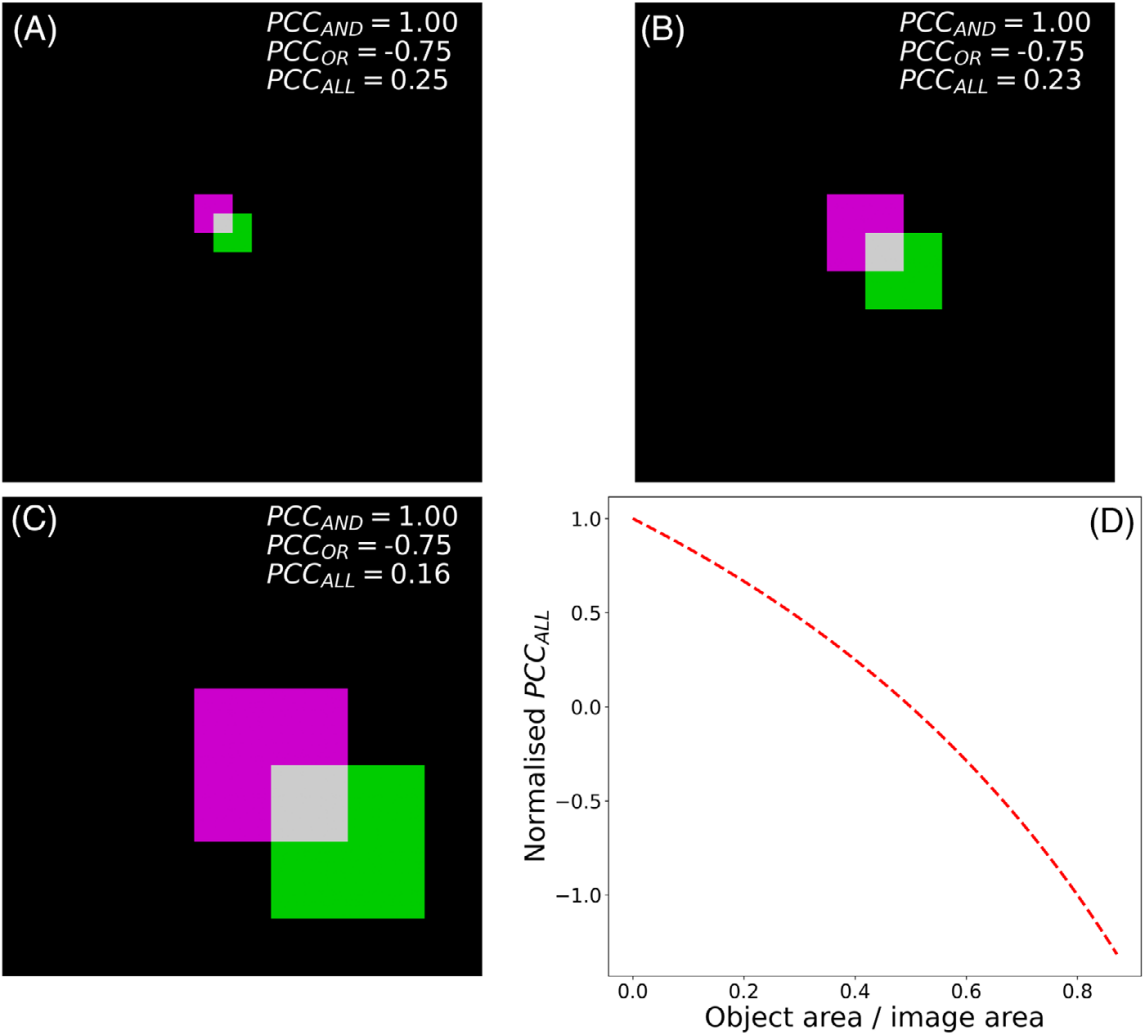
Effect of object size on the PCC values obtained using the different pixel selection criteria. (A)–(C) The overlays of green and magenta images featuring overlapping squares of different sizes. Colocalised areas are shown in white. (D) The relationship between PCC_ALL_ and ratio of object area to image area. The PCC_ALL_ coefficient was normalised by the highest PCC_ALL_ value in the range under consideration (PCC_ALL_ = 0.25). The ratio of object area to image area is calculated as (area of the magenta square + area of the green square)/(total area of the image).

To examine how different object sizes can influence PCC coefficients, we generated images that contain two partially overlapping squares, one on each channel (Figure 2). The sizes of the squares are 80 × 80, 160 × 160, and 320 × 320 pixels in Figure 2A–C, respectively. In all cases, 25% of the area of the magenta square overlaps with 25% of the area of the green square and the total area of each image is 1000 × 1000 pixels. All squares were assigned an arbitrary intensity value of 0.8 and a threshold of 0.1 was used to segment the images for the calculation of the PCC_AND_ and PCC_OR_ coefficients. The PCC_AND_ and PCC_OR_ coefficients are 1.00 and −0.75 for all three figures (i.e., Figure 2A–C), whereas the PCC_ALL_ coefficient varies depending on the sizes of the squares, with values of 0.25, 0.23, and 0.16 (Figure 2A–C) for the objects with 80 × 80, 160 × 160, and 320 × 320 pixels, respectively. To explore the relationship between PCC_ALL_ and object size, we calculated PCC_ALL_ for square sizes covering a wide range from 2 × 2 pixels (PCC_ALL_ = 0.25) to 660 × 660 pixels (PCC_ALL_ = −0.33). All images in this simulation had a size of 1000 × 1000 pixels and the area of overlap between the squares was kept at 25%. The PCC_ALL_ values are normalised by the PCC_ALL_ value for the image with 2 × 2 pixel squares. This analysis illustrates that PCC_ALL_ reduces with object size, while PCC_AND_ and PCC_OR_ are independent of object size (Figure 2A–C). This analysis also shows that, similarly to what was found by examining the images in Figure 1, very different PCC coefficients are sometimes obtained for the same image, depending on the pixel selection criteria employed.

**FIGURE 3:**
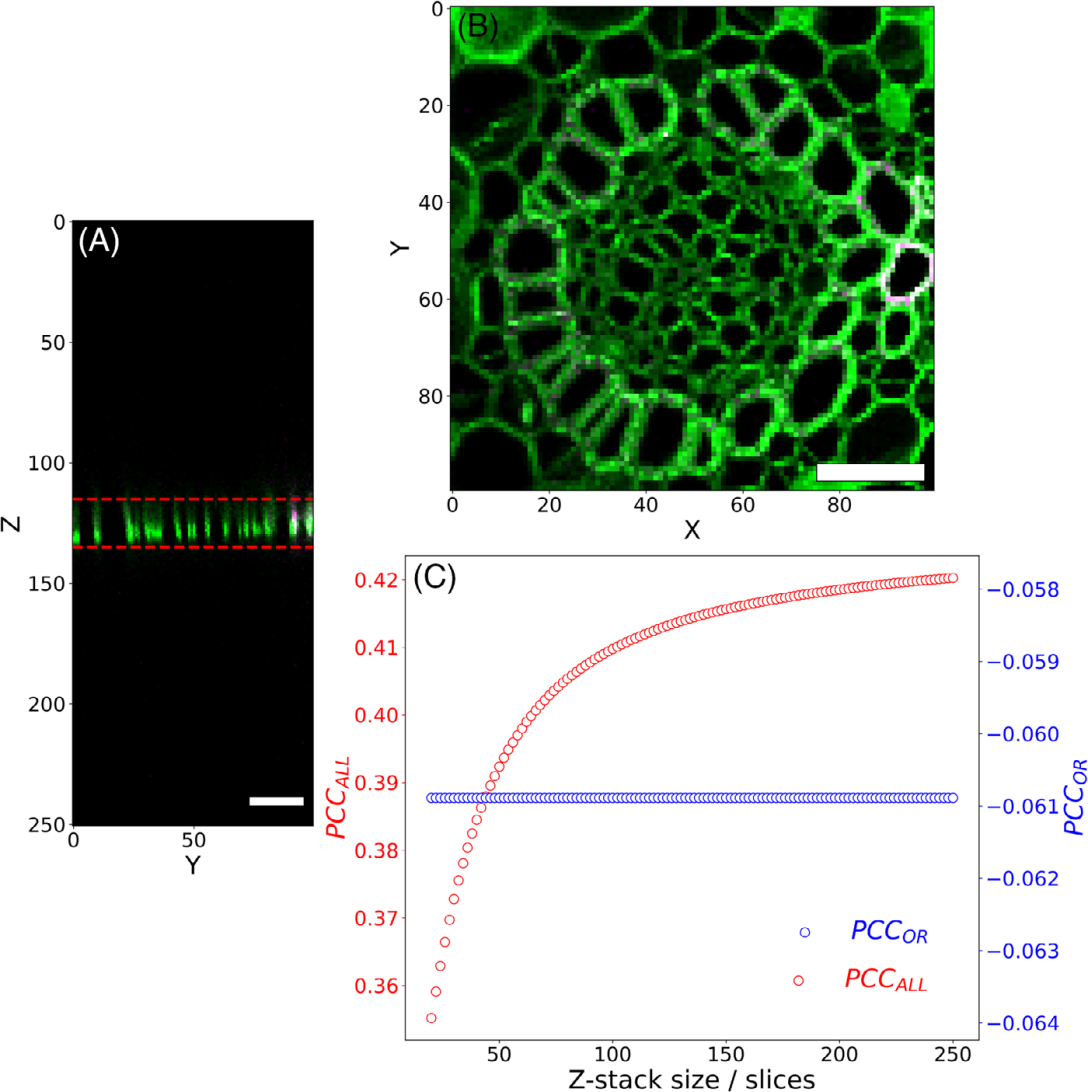
Effect of the z‐range on the PCC coefficients calculated using the different pixel selection criteria. (A) 251‐slice fluorescence microscopy z‐stack of a *Convallaria majalis* sample (Leica Microsystems) stained with fast green FCF (green) and safranin O (magenta). Areas in white are areas of strong colocalisation between the two signals. The horizontal red lines indicate the minimum z‐stack size needed to accurately compute the correlation coefficient of this three‐dimensional image. (B) *XY* display of the central plane (i.e., slice 125) of the *YZ* display shown in (A). Bars, 50 μm. (C) Plot of PCC_ALL_ and PCC_OR_ vs. z‐range. The minimum number of slices is 20 and corresponds to the region encompassed between the two red lines in (A).

Many imaging experiments rely on the collation of information across different planes of section, with the intent to collect as much information as possible across a three‐dimensional structure. Thus, image analysis is frequently performed on projections of z‐stacks. To determine whether uneven fluorescence across different slices influences PCC coefficient calculations, we collected a 251‐slice z‐stack of a thin *Convallaria majalis* stem section (Leica Microsystems). As highlighted by the two red lines in Figure 3A, most of the signal intensity is found in the central 20 slices of the z‐stack, with other slices displaying less and less fluorescence as their distance from the centre of the z‐stack increases. We calculated PCC_ALL_ and PCC_OR_ for different subsets of slice and Figure 3C shows a plot of PCC_ALL_ and PCC_OR_ as a function of the number of z‐slices. It can be seen from Figure 3C that as additional slices above and below the 20 central slices are considered in the calculation, the value of the PCC_ALL_ coefficient progressively increases, with the largest relative changes in PCC_ALL_ taking place as a result of the addition of the first few slices. Gradually, PCC_ALL_ reaches a plateau and becomes comparatively insensitive to the inclusion of more z‐slices. In contrast, PCC_OR_ values are not altered by the inclusion of z‐slices containing little to no fluorescence signal. A similar behaviour is observed for PCC_AND_ (not shown). Thus, the inclusion or exclusion of z‐slices with sparse fluorescence features can have a significant effect on the PCC_ALL_ coefficient of the entire z‐stack.

**FIGURE 4:**
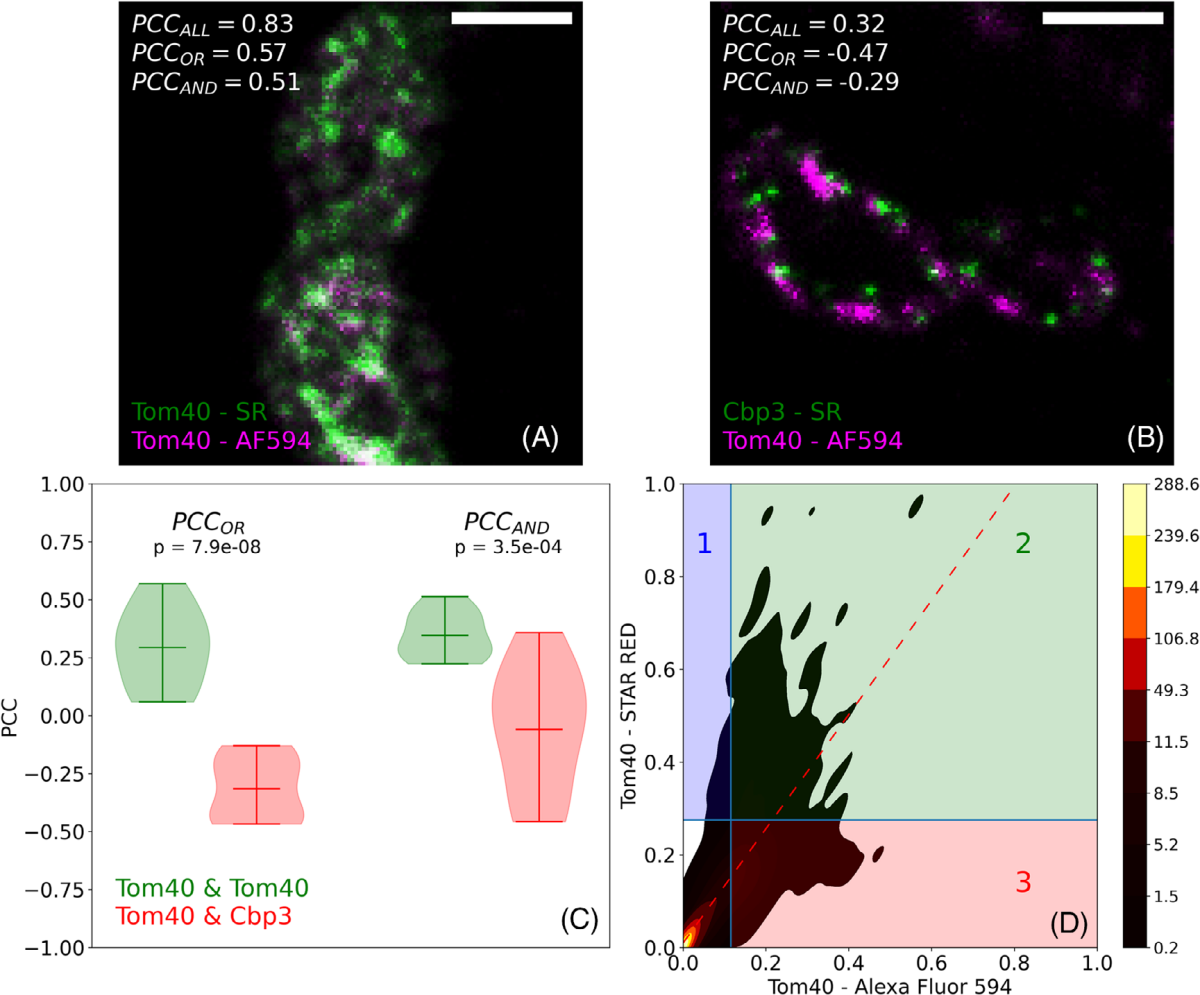
Changes in spatial coincidence between the signals are best examined using PCC_OR_. (A), (B) Dual‐colour Stimulated Emission Depletion (STED) microscopy images of *Saccharomyces cerevisiae* cells. (A) Tom40 as detected using secondary antibodies labelled with either Alexa Fluor 594 (AF594; magenta) or Abberior STAR RED (SR; green). (B) Tom40 and Cbp3, as detected using secondary antibodies labelled with AF594 (magenta) or SR (green), respectively. Bars, 500 nm. (C) Violin plots of PCC_OR_ (left) and PCC_AND_ (right) for Tom40/Tom40 (green) and Tom40/Cbp3 (red). These PCC values were obtained from sets of STED images that comprised 10 images for each protein pair. The *p* values are for Student's *t*‐tests between the Tom40/Tom40 and Tom40/Cbp3 PCC values (left: PCC_OR_; right: PCC_AND_). (D) A kernel density estimation of the cytofluorogram for the image shown in (A). The vertical and horizontal blue lines correspond to the intensity thresholds for the AF594 and SR channels, respectively. The dashed red line is the regression line for all pixels in the image (PCC_ALL_ criterion). PCC_AND_ is calculated using only the pixels in quadrant 2. PCC_OR_ is calculated using the pixels in quadrants 1, 2 and 3. Colour bar, contour levels. PCC_ALL_ includes all pixels in the calculation. The images and data shown in this figure were first published in figure 6 in reference Tameling et al.^27^

To provide an example of a set of images that would be best analysed using PCC_OR_, we made use of a Stimulated Emission Depletion (STED) microscopy dataset first published by Tameling et al.^27^ This dataset comprises dual‐colour STED images of mitochondrial proteins Tom40 and Cbp3 in *Saccharomyces cerevisiae* cells. The proteins were detected via antibodies labelled with Alexa Fluor 594 (AF594) and Abberior STAR RED (SR). Figure 4A shows a SIM image of Tom40 proteins detected using antibodies labelled with AF594 (magenta) or SR (green), whereas Figure 4B shows a SIM image of Tom40 and Cbp3 proteins detected using antibodies labelled with AF594 and SR, respectively. The image shown in Figure 4A (Tom40/Tom40) is meant to serve as a control showing the highest possible colocalisation levels. All PCC values are greater for Figure 4A than for Figure 4B, as expected. However, the difference in PCC_OR_ (Δ_PCC_ = 1.04) is larger than the difference in PCC_AND_ (Δ_PCC_ = 0.8). To test if this observation had general validity, we obtained PCC values for 10 Tom40/Tom40 images and 10 Tom40/Cbp3 images. Violin plots generated using these PCC values can be seen in Figure 4C. The differences in PCC_OR_ between the Tom40/Tom40 and Tom40/Cbp3 images are greater than the corresponding differences in PCC_AND_ with the data for the experimental comparison showing much greater variance for PCC_AND_. The effect size, as computed using Hedges’ *g* test,^28^ was nearly twice as large for the differences in PCC_OR_ (*g* = 3.71) than for the differences in PCC_AND_ (*g* = 1.88). All PCC values were calculated using intensity thresholds obtained by means of the Otsu thresholding method. The kernel density estimation (KDE) shown in Figure 4D corresponds to the cytofluorogram for the image shown in Figure 4A.

**FIGURE 5:**
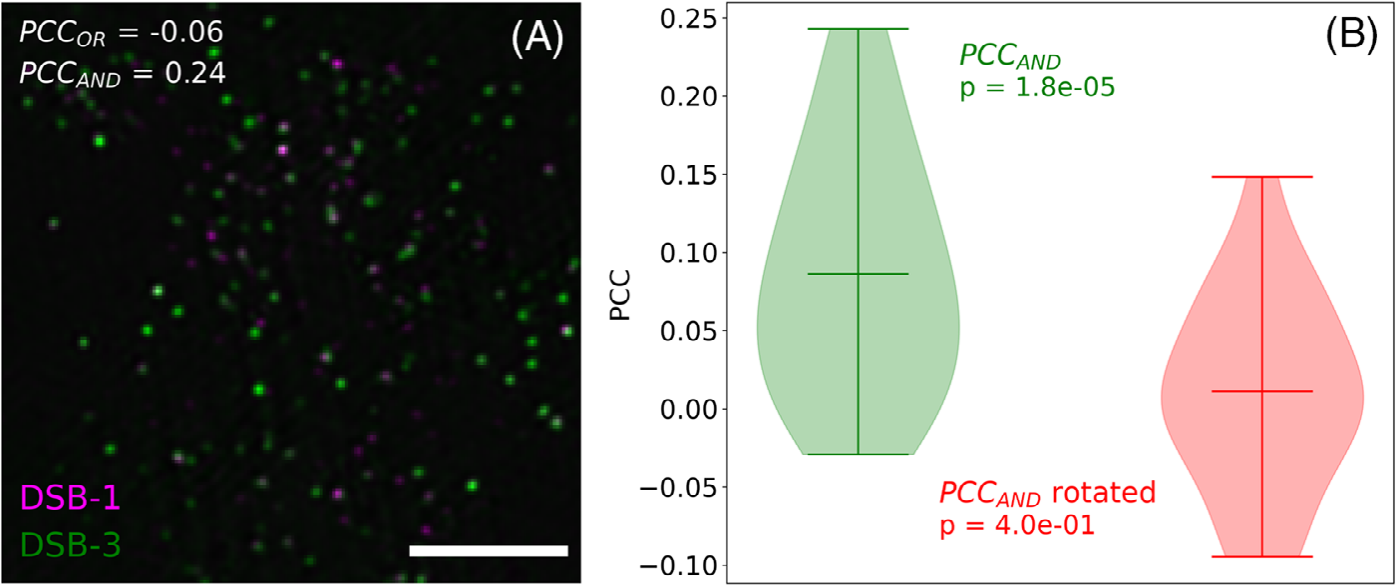
Low levels of colocalisation are best evaluated using PCC_AND_. (A) Structured Illumination Microscopy (SIM) image of a partially spread *Caenorhabditis elegans* nucleus featuring DSB‐1 (magenta) and DSB‐3 (green) proteins labelled with fluorophore‐conjugated antibodies (DBS‐1: Alexa Fluor 488; DBS‐3: Alexa Fluor 555). Bar, 2 μm. (B) PCC_AND_ violin plots for DSB‐1 and DSB‐3 obtained by analysing a set of SIM images (*N* = 24) of partially spread *C. elegans* nuclei images of which (A) is a representative example. The plot labelled as ‘rotated’ corresponds to the PCC_AND_ values obtained after rotating all the DBS‐1 images in the set by 90 degrees. The *p* values are for one‐sample Student's *t*‐tests between the PCC_AND_ values and a hypothesised mean of zero (i.e., no correlation). The images used in this figure were downloaded from the EMBL‐EBI BioImage Archive^26^ and were first published in figure 5 in reference Hinman et al.^29^

PCC_OR_ can fail to detect low levels of colocalisation. The Structured Illumination Microscopy (SIM) image of a partially spread *Caenorhabditis elegans* nucleus displayed in Figure 5A shows low levels of colocalisation between the two proteins under study, DSB‐1 and DSB‐3, as evinced from the scarcity of white pixels. For this image, only PCC_AND_ adopts a positive value (PCC_AND_ = 0.24), with PCC_OR_ taking a negative value instead (PCC_OR_ = −0.06). The violin plots in Figure 5B show the PCC_AND_ values obtained for a set of 24 DBS‐1/DBS‐3 SIM images of partially spread nuclei. The plot denoted as ‘rotated’ corresponds to PCC_AND_ values obtained after rotating the DBS‐1 images by 90 degrees. All DBS‐1/DBS‐3 images used here were first published by Hinman et al.^29^ The PCC_OR_ values for the set of 24 unrotated SIM images were all negative (Figure S2). All PCC_OR_ and PCC_AND_ values were calculated using intensity thresholds obtained via the Otsu thresholding method. One‐sample Student's *t*‐tests were consistent with only PCC_AND_ agreeing with the conclusion that DBS‐1 and DBS‐3 colocalise in *C. elegans* nuclei, as shown by Hinman et al.^29^ by means of an object‐based colocalisation approach.

## 4 DISCUSSION

Our analysis identifies that by applying different pixel selection criteria to the calculation of the PCC coefficient we are in fact measuring different image properties (Figure 1). PCC_ALL_ is a coefficient that is influenced not only by the spatial cooccurrence of the two fluorescence signals and the proportionality of their intensities, but also by the ratio of foreground to background pixels. This ratio depends on factors such as object size, object shape, object density and even the size of the image in which the object is present. These factors are entirely unrelated to the biological quantity that fluorescence colocalisation intends to quantify, which is the degree of association of two fluorescently labelled molecules.

PCC_OR_, on the other hand, is a more restrictive pixel selection criterion than PCC_ALL_. Therefore, comparatively fewer pixels are included in the calculation of PCC_OR_ than in the calculation of PCC_ALL_. By excluding background pixels from the calculation, PCC_OR_ more accurately reflects the biological quantity of interest. Since pixels that are above one of the thresholds, even if they are not above both thresholds, have an impact on the calculation, the PCC_OR_ coefficient quantifies both the extent to which the signals cooccur in the image and the relationship that the two fluorescence intensities show with respect to each other. In other words, PCC_OR_ is influenced both by signal cooccurrence and by signal proportionality. A simultaneous dependence on these two factors is perhaps what most researchers would expect from a coefficient that is meant to represent the extent to which two biomolecules interact with each other.

PCC_AND_ is even more restrictive in terms of pixel selection than PCC_OR_. Since in the case of PCC_AND_ only pixels that are above both thresholds are included in the calculation, signal cooccurrence is a property of all the pixels being considered. Therefore, signal cooccurrence does not influence the magnitude of PCC_AND_, and all the variability is attributable to signal proportionality or the lack thereof. Because the pixels selected for the calculation have relatively high intensities in both channels, PCC_AND_ is more likely to detect small levels of signal colocalisation. For instance, only PCC_AND_ adopts a moderately positive value for the image shown in Figure 1D, with PCC_ALL_ and PCC_OR_ adopting null and negative values, respectively, for the very same image. This is noteworthy because the proteins that are showcased in the image, BRCA1 and Smad3, do interact with each other in the nucleus, as has been shown using other techniques.^25^, ^30^, ^31^

It is evident from Figure 2 that PCC_AND_ and PCC_OR_ show distinctly different behaviours. PCC_AND_ overemphasises small regions of colocalisation, which makes it more likely to adopt positive values, whereas PCC_OR_ overemphasises the absence of signal cooccurrence, which makes it more likely to adopt negative values. Quite surprisingly, the PCC_ALL_ values depicted in Figure 2D span both negative and positive values, even when signal proportionality and spatial overlap remain invariant. This raises concerns about the interpretability of the PCC_ALL_ coefficient because this parameter could, in principle, take values indicative of mutual exclusion or near perfect colocalisation for samples in which the degree of biomolecular interaction is the same. It may then come as an even bigger surprise that, to our knowledge, the PCC_ALL_ pixel selection criterion is one of the most, if not the most, widely implemented criterion for the calculation of the PCC coefficient. The reason for this is that this pixel selection criterion does away with the need for a suitable threshold value to distinguish between foreground and background. Finding such a threshold value is not trivial and the choice of the thresholding algorithm can also have a profound effect on the value of the PCC coefficient. If the threshold value is found visually on an image per image basis, the results become poorly reproducible and largely arbitrary. If an automatic thresholding algorithm is utilised (e.g., Otsu, Li, Minimum, Isodata), there is the possibility that in dimmer images large swathes of background will be identified as foreground, the reason being that histogram‐based thresholding methods always split the image into foreground and background, even in the case of images that consist solely of autofluorescence or unspecific staining.

Our analysis here also highlights the importance of collecting or analysing images that contain information. Figure 3 demonstrates that the adoption of the PCC_ALL_ pixel selection criterion can strongly influence PCC values calculated for three‐dimensional data (i.e., z‐stacks). As can be seen in Figure 3C, the largest relative changes to PCC_ALL_ result from the inclusion or exclusion of just a few z‐slices above and below the object of interest. Thus, to keep the PCC values comparable in between images, it is necessary to acquire z‐stacks that contain only as many z‐slices as are necessary to fully capture the object of interest. In practise, however, it seems more appropriate that, when three‐dimensional data need to be acquired, researchers should rely on pixel selection criteria that are independent of the foreground to background ratio, namely, PCC_AND_ and PCC_OR_, which remain constant regardless of the number of slices included in the calculation of this coefficient. Although the potential issue arising from the dependence of PCC_ALL_ on the foreground to background ratio is raised here in relation to three‐dimensional data, it also applies to datasets with higher dimensionality. For instance, if colocalisation is monitored over time and the acquisition of a time‐series is consequently in order, researchers should be mindful that the acquisition of time points for which the images are largely empty, for example, due to the photobleaching of the fluorophores, could potentially impact the PCC coefficient if the PCC_ALL_ pixel selection criterion is used.

On occasion, it is necessary to quantify differences in signal cooccurrence for two experimental conditions. PCC_OR_ is more suited to this task than PCC_AND_. An illustrative example is provided by the analysis of images that were first published by Tameling et al.^27^ These images, which were obtained using STED microscopy, show the spatial distribution of Tom40 and Cbp3, a pair of mitochondrial proteins. Cryoimmunogold electron microscopy studies have revealed that Tom40 and Cbp3 are located in close proximity in the mitochondrial membrane.^32^, ^33^ Figure 4B shows a representative Tom40/Cbp3 STED image, which features these two proteins as detected in yeast mitochondria using fluorescently labelled antibodies. Although Tom40 and Cbp3 are expected to colocalise with each other, their spatial distributions in Figure 4B do not show the same degree of overlap as that found in a positive control image, Figure 4A, in which the same protein, Tom40, was detected using two different fluorescently labelled antibodies. To quantify what is basically a difference in signal cooccurrence between Tom40/Cbp3 and the positive control, Tom40/Tom40, PCC_ALL_ and PCC_OR_ values were calculated for a set of images that contained ten images for each protein pair. The violin plots of these PCC values (Figure 4C) suggest that PCC_OR_ is more sensitive than PCC_AND_ to differences in spatial coincidence between the signals. Hedges’ g tests supported this, showing a much larger effect size for PCC_OR_ than for PCC_AND_.

The cytofluorogram shown in Figure 4D provides a visual representation of the sets of pixels that are included in the PCC_ALL_, PCC_OR_ and PCC_AND_ calculations. PCC_ALL_ involves all pixels in the cytofluorogram; PCC_OR_ is restricted to pixels in quadrants 1, 2 and 3; lastly, PCC_AND_ entails only those pixels in quadrant 2. Considering this, it is easy to see why in images with a low degree of spatial cooccurrence between the signals, where most pixels are in quadrants 1 and 3, the slope of the regression line can easily take negative values, as exemplified in Figure S1B.

As previously mentioned, low levels of colocalisation are more likely to be detected using PCC_AND_, rather than PCC_OR_. A good example of this can be found in the analysis of the SIM images first published by Hinman et al.^29^ These are 24 images of partially spread *C. elegans* nuclei depicting DBS‐1 and DBS‐3 proteins detected via fluorescently labelled antibodies. A representative example of these images is shown in Figure 5A. As shown in the image itself, PCC_OR_ takes a negative value in this case (PCC_OR_ = −0.06), suggesting no colocalisation between DBS‐1 and DBS‐3. The remaining 23 images in the dataset also adopt negative PCC_OR_ values (Figure S2) and, therefore, fail to detect any meaningful colocalisation between the probes. By contrast, nearly all the PCC_AND_ values are above zero (Figure 5B). To determine if there are significant differences between these PCC_AND_ values and those that would be expected in the absence of colocalisation, we deployed both statistical and randomisation‐based analyses. Statistical analysis compares the mean of PCC values with zero, which is the value expected in the absence of any correlation between the signals. This comparison can be conducted using a one‐sample Student's *t*‐test.^34^, ^35^ In our example data, only the PCC_AND_ values obtained using the unrotated image dataset were found to significantly differ from the hypothesised mean (*p* value = 1.8 × 10^−5^). Randomisation‐based analysis relies on comparing the PCC values obtained for an image of interest with PCC values obtained after either rotating or scrambling this image.^36^ For our example data, a two‐sample Student's *t*‐test to compare the PCC_AND_ values for the unrotated and rotated datasets suggests that the rotated data differs from the unrotated data (*p* value = 6.3 × 10^−4^). Therefore, both the statistical and the randomised analyses support the idea that PCC_AND_ can detect low colocalisation levels.

Here, we aim to encourage users to understand the analyses they perform on their data, and the limitations of each calculation. We also aim to encourage software engineers to make the information regarding which calculations are used in different analysis tools readily accessible. This is of utmost importance because different pixel selection criteria result in PCC coefficients that report on different properties, but information about the pixel selection criterion can sometimes only be obtained by reverse engineering, or by a considerable knowledge of the programming language in which the software is written. In the Supporting Information, we include a list of popular colocalisation software packages and the pixel selection criteria that are applied in each case.

Since PCC is a coefficient that has found widespread use in the community, it is not surprising that we are not the first to point out that the exclusion of background pixels from the calculation of this coefficient is in most cases something to be desired. For instance, in their 2010 paper, Barlow et al.^37^ argued for the use of a thresholded PCC, as opposed to a PCC that encompasses all pixels in the image. They made the case that to avoid false positives, and for the PCC coefficient to accurately describe the relationship between a pair of signals, background pixels should not form part of the PCC calculation. Further, the difficulties associated with thresholding prompted Costes et al.^38^ to develop a thresholding method that incorporates the calculation of the PCC coefficient into it. In Costes’ method the thresholds are initialised to high values, and the PCC for the pixels below the thresholds is computed. The thresholds are then lowered by a certain amount and the PCC for the pixels below the thresholds is computed again. The algorithm proceeds in this manner until the PCC for the pixels below the thresholds is either null or negative. The first threshold values for which this condition is fulfilled are chosen as the preferred threshold values and the PCC for the pixels above the thresholds is calculated.^38^ Although Costes’ method has proven very useful under many conditions,^39^, ^40^, ^41^, ^42^, ^43^, ^44^ some authors have pointed out that this method sometimes find images with high labelling densities difficult to deal with, and that it also struggles with images that feature many more objects in one channel than in the other.^45^

Notwithstanding the potential challenges in the search for a suitable automatic thresholding method, it is worth noting that great progress has been made in the past few years in the field of image segmentation. Nowadays, machine learning methods, such as those that rely on the use of convolutional neural networks, can provide reliable segmentation results without being affected by the limitations of histogram‐based segmentation methods.^46^, ^47^, ^48^, ^49^, ^50^ For this reason, in the future we expect thresholding in the context of colocalisation analysis to be less challenging, and more widely implemented by the scientific community.

## 5 CONCLUSIONS

After having examined the three possible pixel selection criteria that can be applied to the calculation of PCC, we find that PCC_ALL_ is the most problematic of them, due to its dependence on the foreground to background ratio. Under most circumstances, PCC_ALL_ is best avoided. We appreciate that when designing a software solution for a wide user base, it is difficult, if not impossible, to adopt an automatic thresholding method that can suit all kinds of images. Although PCC_ALL_ can provide reliable results for sets of two‐dimensional images with similar foreground to background ratios, the advent of modern automatic thresholding methods based on machine learning algorithms might eventually remove the obstacles associated with thresholding to such an extent that even for this kind of images PCC_OR_ and PCC_AND_ will be the best possible options. When it comes to the choice between PCC_OR_ and PCC_AND_, the former is more suited to circumstances under which evaluating the cooccurrence of the signals is of paramount importance, whereas the latter is more suited to the detection of small degrees of colocalisation between biomolecules. However, of primary importance is that users understand the different PCC coefficients and draw conclusions based on the power and limitations of each coefficient, to best interpret the biological inferences contained in their data.

## Supporting information

Supporting Information
