# Supplementary material for: On the pixel selection criterion for the calculation of the Pearson's correlation coefficient in fluorescence microscopy
Source: J Microsc. 2024 Feb 13;297(3):304–15. doi: 10.1111/jmi.13273 (PMC11808421; doi:10.1111/jmi.13273)
Supplement: Supplementary file 1 — Supporting Information [file JMI-297-304-s001.docx]

**SUPPORTING INFORMATION**


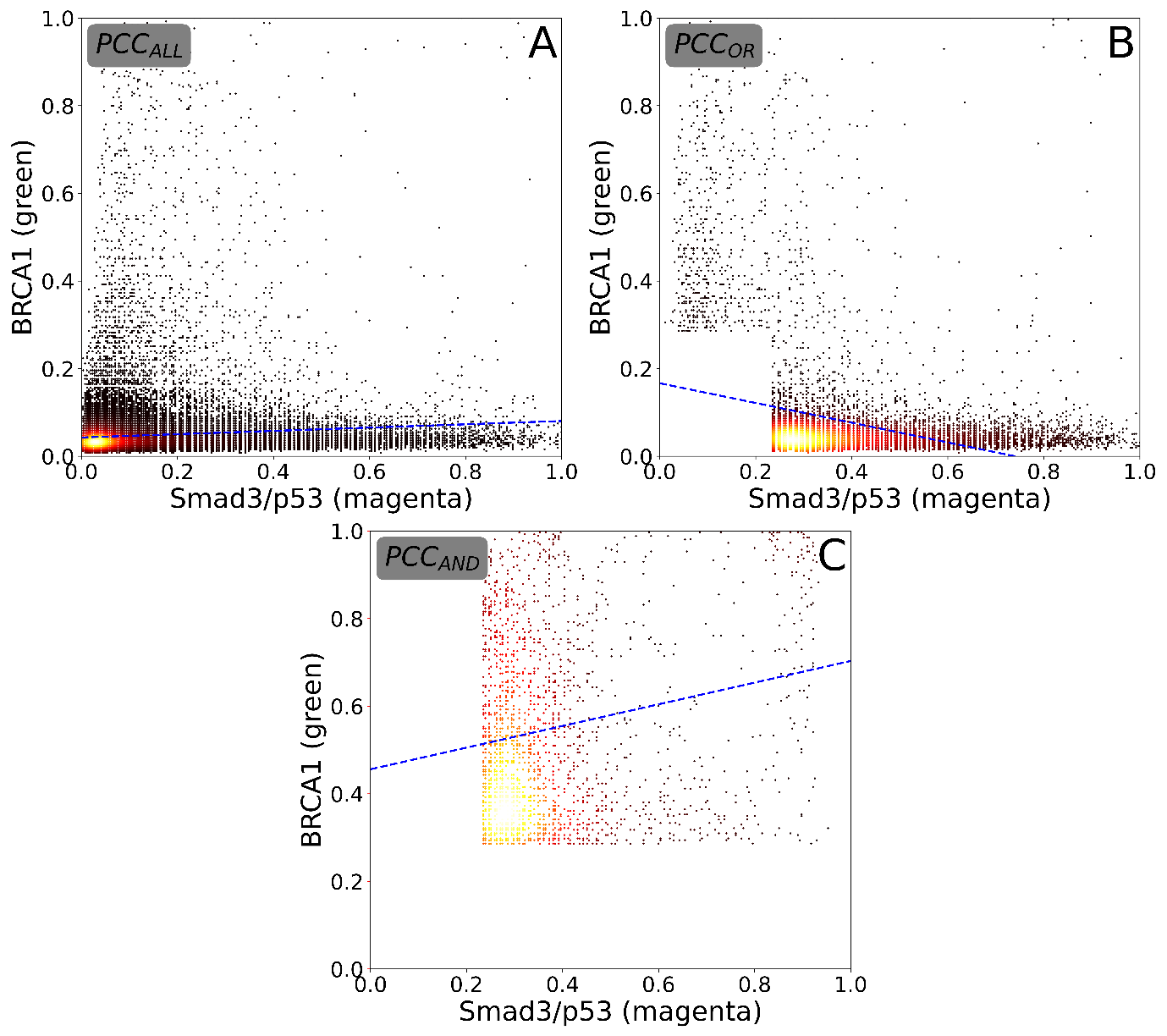


Figure S1. The choice of the pixel selection criterion has a strong impact on the set of data points used for the calculation of the PCC. (A), (B), and (C) show the cytofluorograms obtained after applying the PCC_ALL_, PCC_OR_, and PCC_AND_ pixel selection criteria, respectively, to the image shown in figure 1D. To aid visualisation, (A) displays only 2% of the image pixels, whereas (B) displays only 10% of the pixels that remain after the implementation of the PCC_OR_ pixel selection criterion. In both cases, the displayed pixels were randomly selected. (C) displays all of the pixels that remain after the implementation of the PCC_AND_ pixel selection criterion. The dashed blue lines represent the best linear fits of the displayed data points.


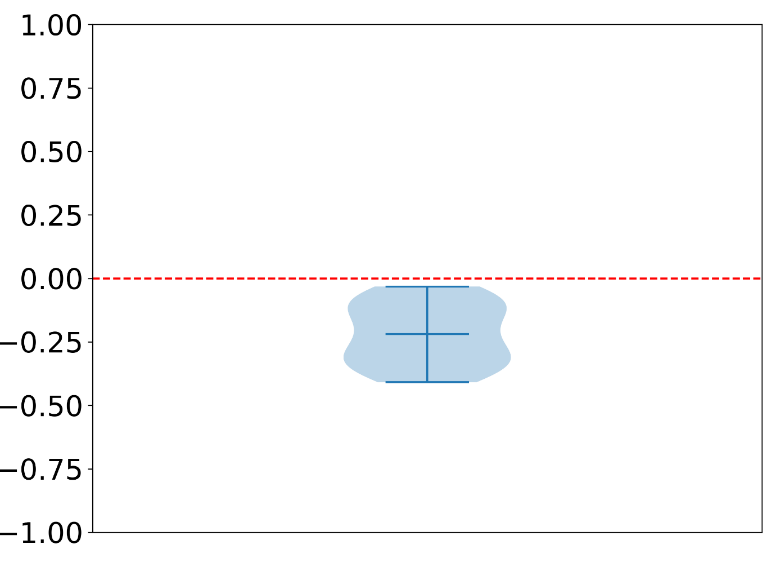


Figure S2. PCC_OR_ violin plots for DSB-1 and DSB-3 obtained by analysing a set of SIM images (N = 24) of partially spread *C. elegans* nuclei images of which (figure 5A) is a representative example. Red line, PCC = 0.

*Non-exhaustive list of software solutions that implement the pixel selection criteria mentioned in the main text of the article.* Huygens deconvolution (Scientific Volume Imaging, The Netherlands, <http://svi.nl>), LAS X (Leica Microsystems), and ImageJ plugins JACoP^1^ and Colocalize…^2^ make use of the PCC_ALL_ pixel selection criterium or a variation thereof for some of the PCC values they generate. The ImageJ plugins Colocalization Threshold^2^ and the implementation of the Costes’ automatic thresholding algorithm in JACoP^1^ make use of the PCC_AND_ pixel selection criterium. Finally, the ImageJ plugin Coloc2^2^ makes use of the PCC_OR_ pixel selection criterium. MetaMorph (Molecular Devices), and the colocalisation module in Zen (Carl Zeiss) implement both the PCC_ALL_ and the PCC_AND_ algorithms.

**REFERENCES**

1. Bolte S, Cordelières FP. A guided tour into subcellular colocalization analysis in light microscopy. J Microsc-Oxford. 2006;224:213-32.

2. Schindelin J, Arganda-Carreras I, Frise E, Kaynig V, Longair M, Pietzsch T, et al. Fiji: an open-source platform for biological-image analysis. Nat Methods. 2012;9(7):676-82.
